# Dose-dependent effects of sodium bicarbonate supplementation combined with sprint interval training on performance adaptations in basketball players: a randomized placebo-controlled trial

**DOI:** 10.1080/15502783.2026.2735090

**Published:** 2026-10-09

**Authors:** Xinyun Wang, Xinlei Wang

**Affiliations:** a School of Physical Education, Huzhou Normal University, Huzhou, People's Republic of China; b School of Physical Education, Ningbo University, Ningbo, Zhejiang, People's Republic of China

**Keywords:** Sodium bicarbonate, sprint interval training, buffering capacity, anaerobic power, basketball performance, dose–response

## Abstract

**Background:**

Sprint interval training (SIT) effectively improves physical performance in team-sport athletes, while sodium bicarbonate (Sb) supplementation may enhance training adaptations. However, the dose-dependent effects of Sb during SIT remain unclear in basketball players. Therefore, this study examined the effects of two Sb doses combined with SIT on physical performance adaptations in basketball players.

**Methods:**

Forty trained male basketball players (age: 21.6 ± 2.0 years, height: 185.4 ± 3.5 cm, body mass: 84.1 ± 4.4 kg) were recruited and randomly assigned to four groups: SIT + 0.3 g·kg^−1^ Sb (*n* = 10), SIT + 0.45 g·kg^−1^ Sb (*n* = 10), SIT + placebo (PL) (*n* = 10), and control (CON) (*n* = 10). The intervention lasted four weeks during the preparatory phase of the season. Participants in the training groups performed SIT three times per week in addition to their regular basketball training. The SIT protocol consisted of four sets of ten 5-second maximal sprints, with 20 seconds of active recovery between repetitions and 2 minutes of rest between sets. All 40 enrolled participants successfully completed the 4-week intervention, resulting in a 0% dropout rate. Performance assessments conducted before and after the intervention included countermovement vertical jump (CMVJ), standing long jump (SLJ), 20 m sprint, Illinois change-of-direction speed (CODS), Yo-Yo Intermittent Recovery Test Level 1 (Yo-Yo IR1), and Wingate anaerobic power (peak power output [PPO] and mean power output [MPO]).

**Results:**

All training groups showed significant improvements in physical performance variables over time (*p* < 0.05). Significant group by time interaction effects were observed for all performance variables (*p* ≤ 0.001), indicating that the SIT groups showed more gains compared with the CON group. Both Sb supplementation groups demonstrated greater improvements than the SIT + PL group in CMVJ, SLJ, sprint performance, CODS, Yo-Yo IR1 distance, PPO, and MPO (*p* < 0.05). Furthermore, the SIT + 0.45 g·kg^−1^ Sb group exhibited significantly greater performance enhancements than the SIT + 0.3 g·kg^−1^ Sb group across all measured variables.

**Conclusion:**

Four weeks of SIT improved performance in trained basketball players, with Sb supplementation providing additional dose-dependent benefits (0.45 > 0.3 g⋅kg^−1^). While the higher dose yielded superior adaptations, its practical application should be balanced against potential gastrointestinal discomfort and individual tolerance to ensure long-term athlete compliance.

## 1. Introduction

Basketball is a high-intensity intermittent sport characterised by repeated bouts of sprinting, jumping, accelerations, and rapid changes of direction interspersed with short recovery periods [1,2]. These repeated explosive movements impose substantial demands on both aerobic and anaerobic energy systems [3]. During high-intensity exercise, accelerated glycolytic metabolism leads to the accumulation of hydrogen ions and lactate in the muscle and blood, contributing to metabolic acidosis [4]. This disturbance in acid–base balance can impair muscle contraction, reduce power output, and accelerate fatigue [5]. Given that managing this metabolic acid-base disturbance is vital for maintaining performance during repeated bouts, identifying integrated training and nutritional strategies that can bolster buffering systems is essential [5]. Consequently, training strategies (e.g. sprint interval training) and nutritional interventions (e.g. sodium bicarbonate) that enhance buffering capacity and improve tolerance to high-intensity exercise are of considerable interest in sports performance research [6,7].

Optimising conditioning in team sports requires a delicate balance between sport-specific technical demands and physiological capacity. Small-sided games (SSGs) are a fundamental training modality in this regard, as they simultaneously integrate technical, tactical, and physiological demands, offering a more ecological training stimulus [8]. Recent evidence suggests that optimising training bout durations in SSGs is critical for balancing technical proficiency and metabolic load [8]. Conversely, sprint interval training (SIT) offers a highly controlled, repetitive stimulus, which serves as a potent supplementary approach to bridge specific conditioning needs—particularly when aiming for rapid increases in anaerobic capacity—that may not be fully addressed by SSGs alone [6].

SIT has been widely recognised as an efficient method for inducing significant physiological adaptations within relatively short training durations [3,9–11]. By utilising repeated maximal or near-maximal efforts separated by brief recovery intervals, SIT effectively enhances both aerobic and anaerobic systems [12,13]. While SIT is a potent driver of metabolic efficiency and power output [3,9–12], it is essential to contrast its adaptations with other explosive conditioning modalities. For instance, whereas SIT predominantly targets metabolic capacity, modalities such as plyometric training combined with change-of-direction (COD) sprints are fundamental for enhancing neuromuscular power and court agility [14]. Unlike SIT’s primary focus on high-intensity metabolic load, plyometric and COD-focused training specifically target neural drive, stretch-shortening cycle efficiency, and multi-directional force production [14]. Recognising these distinct training stimuli—metabolic versus neuromuscular—is crucial for a comprehensive conditioning overview in team sports. For basketball players, whose performance relies on a combination of aerobic capacity to sustain efforts and explosive neuromuscular power to execute jumping and sprinting actions throughout the game [15], SIT serves as a critical component in bridging the gap between general conditioning and high-intensity match demands.

Alongside training interventions, nutritional ergogenic aids have been widely investigated as complementary strategies to enhance exercise performance and training adaptations [16,17]. Sodium bicarbonate (Sb) is one of the most extensively studied extracellular buffering agents because it increases blood bicarbonate concentration and enhances the body’s ability to buffer hydrogen ions produced during intense exercise [18–24]. The commonly recommended ergogenic dose is approximately 0.3 g·kg^−1^ of body mass, which has been consistently shown to improve performance in high-intensity activities such as jumping and sprinting abilities as well as COD [19]. By enhancing extracellular buffering capacity, Sb may delay fatigue during repeated maximal efforts and potentially augment training-induced adaptations [23,24].

However, despite the well-established ergogenic effects of Sb, the dose-dependent impact of Sb supplementation when combined with SIT remains largely unexplored in trained basketball players, highlighting the need to determine whether higher dosages provide superior training adaptations.

During short and intensive phases of the annual training plan, such as specific preparatory or pre-competitive periods, athletes are often exposed to concentrated blocks of high-intensity training designed to rapidly enhance physical performance [25]. These phases are characterised by substantial metabolic stress and repeated exposure to anaerobic exercise, which may increase the demand for effective acid–base regulation [4,5]. Under such conditions, a greater buffering capacity could support higher training quality and potentially enhance adaptations in both aerobic and anaerobic performance variables [26–29].

While 0.3 g·kg⁻^1^ of Sb is established as the standard ergogenic threshold, the “ergogenic ceiling” remains a subject of ongoing debate, particularly in the context of chronic high-intensity interval training (i.e. SIT) [18,19,30]. From a mechanistic perspective, a critical trade-off exists: while higher doses of Sb significantly amplify extracellular buffering capacity and the neutralisation of metabolic acidosis, they concomitantly increase the risk of gastrointestinal distress, which can exert an ergolytic effect by compromising participant comfort and focus [19]. To navigate this trade-off, the current protocol utilised a split-dosing strategy designed to attenuate the osmotic load and prolong the absorption window, thereby maximising bicarbonate availability while minimising gastrointestinal (GI) perturbation. Based on the premise that chronic SIT induces a cumulative metabolic acid-base stress exceeding that of acute single-bout exercise [30], we hypothesised that the 0.45 g·kg⁻^1^ dose would provide a superior physiological environment for performance adaptations including anaerobic power, aerobic fitness, jumping, sprinting and COD speed. We further posited that, through careful management of GI tolerance, this higher dose would elicit a greater dose-dependent performance enhancement than the standard 0.3 g·kg⁻^1^ dose, suggesting that the ergogenic potential of Sb in chronic training is likely underestimated when limited to conventional dosages.

## 2. Materials and methods

### 2.1. Participants

Forty trained young male basketball players volunteered to participate in this study during the preparation phase of their annual training cycle. All participants were active competitors at the local club level, with a minimum of five years of systematic basketball training experience. To ensure a consistent training status across the sample, participants were required to be involved in at least four organised training sessions per week (approximately 90-120 minutes per session) in addition to their regular competitive schedule [31]. The players were regularly engaged in structured team training sessions and were familiar with high-intensity conditioning protocols. The participants were randomly assigned to experimental groups receiving different Sb dosages during the SIT programme (Table 1). Prior to participation, all athletes were informed about the purpose, procedures, and potential risks of the study, and written informed consent was obtained from all participants. The study protocol was conducted in accordance with the ethical standards of the Declaration of Helsinki and was approved by the institutional research ethics committee of the Huzhou Normal University. Participants were screened to ensure that they were free from cardiovascular, metabolic, or musculoskeletal disorders and had not consumed buffering supplements or other ergogenic aids for at least four weeks prior to the study. They were also instructed to maintain their normal diet and training routines throughout the experimental period and to refrain from consuming caffeine or other stimulants before testing or training sessions.

**Table 1.** Participants' characteristics (mean ± SD).

Groups	Age (y)	Height (cm)	Body mass (kg)	Basketball training experience (y)
SIT + 0.3 g Sb	21.4 ± 2.2	186.3 ± 3.9	84.1 ± 5.2	8.3 ± 2.2
SIT + 0.45 g Sb	21.6 ± 1.8	185.0 ± 3.7	84.6 ± 4.3	7.8 ± 1.7
SIT + PL	22.0 ± 1.9	186.2 ± 2.4	82.3 ± 2.7	7.4 ± 1.5
CON	21.5 ± 2.1	184.2 ± 4.1	85.5 ± 5.4	8.2 ± 1.9

SIT: sprint interval training, Sb: sodium bicarbonate, PL: placebo, CON: control.

### 2.2. Sample size estimation and randomisation

The required sample size was estimated a priori using G*Power software (version 3.1.9, University of Düsseldorf, Düsseldorf, Germany). The calculation was based on a repeated-measures analysis of variance (ANOVA) with a within–between interaction design, evaluating changes in key performance variables such as the vertical jump. Assuming a medium effect size (f = 0.25), which is considered a conservative and practically meaningful threshold for trained basketball populations—where large performance adaptations are less frequent over short-term (4-week) intervention periods—a statistical power of 0.80 and an alpha level of 0.05 were adopted. Based on these parameters, the minimum required sample size was estimated at 36 participants [9]. To account for potential dropouts or incomplete data, a total of 40 participants were recruited and randomly allocated into four groups (*n* = 10 per group) using a computer-generated sequence. The groups consisted of: (1) SIT with 0.3 g·kg⁻^1^ Sb supplementation (SIT + 0.3 g Sb, *n* = 10), (2) SIT with 0.45 g·kg⁻^1^ Sb supplementation (SIT + 0.45 g Sb, *n* = 10), (3) SIT with placebo (SIT + PL, *n* = 10), and (4) a control group (CON, *n* = 10) that continued their regular basketball training without supplementation. Participants were randomly allocated into four groups using a computer-generated randomisation procedure. Randomisation was performed by an independent researcher who was not involved in data collection to ensure allocation concealment and minimise potential bias.

### 2.3. Study design

This study employed a randomised controlled experimental design to examine the effects of Sb supplementation with varying dosages combined with SIT on performance adaptations in trained basketball players during a short-term preparatory training phase. Participants completed a series of performance assessments before (pre-test) and after (post-test) a four-week training intervention. Prior to the intervention, all participants attended a familiarisation session during which the testing procedures were explained and practiced to minimise potential learning effects. Baseline testing was conducted over three separate days with at least 48 hours between sessions to reduce fatigue. The testing battery was conducted over three separate days to minimise fatigue and ensure reliable performance measurements. On the first day, participants completed tests of explosive power and speed, including the countermovement vertical jump (CMVJ), standing long jump (SLJ), 20-m sprint, and the Illinois change of direction speed (CODS) test. On the second day, aerobic endurance performance was assessed using the Yo-Yo Intermittent Recovery Test Level 1 (Yo-Yo IR1). On the third day, anaerobic power performance was evaluated using the Wingate anaerobic power test performed on a cycle ergometer. Sb or PL supplementation was administered according to group allocation throughout the days of weekly training session (i.e. four weeks). All training sessions were supervised by the research team to ensure adherence to the prescribed protocol. Following the intervention period, all participants repeated the same battery of tests under identical conditions and at the same time of day as the baseline assessments (i.e. afternoon, 16:00-18:00) to evaluate training-induced adaptations in performance variables. Post-intervention testing was performed 72 hours after the final Sb ingestion to ensure the dissipation of acute buffering effects. As blood bicarbonate levels generally return to baseline within 24 hours, this washout period allowed post-test results to reflect training-induced adaptations rather than acute supplementation effects [18,20].

### 2.4. Supplementation

Participants assigned to the Sb supplementation groups consumed Sb at doses of 0.3 g·kg^−1^ body mass or 0.45 g·kg^−1^ body mass (Nutricost, Vineyard, UT) [19]. Individual doses were calculated based on each participant’s body mass. To minimise the likelihood of gastrointestinal discomfort and improve tolerability, the total daily dose was divided into five equal servings. All athletes were instructed to consume either the supplement or the PL five times per day at standardised times (08:00, 12:00, 16:00, 20:00, and 24:00), with each dose mixed in approximately 200 mL of water or juice. The PL group received maltodextrin administered in the same dosing schedule and volume as the supplementation groups. Both Sb and PL were encapsulated in identical opaque capsules to maintain double-blind conditions. Neither the participants nor the investigators involved in training supervision or data collection were aware of the capsule contents until completion of the study.

Compliance with the supplementation protocol was monitored through weekly adherence logs, verbal confirmation during training sessions, and the return of empty supplement containers at the end of each week. Supplement packages were distributed and counted weekly to verify adherence throughout the intervention period. To ensure strict double-blind conditions, all supplements were administered in identical, opaque capsules. Given the inherent differences in bulk density and volume between Sb and the PL, the supplementation protocol was standardised to achieve visual and physical uniformity. Participants ingested about five size 000 capsules per dose, adjusted individually based on body mass. By matching all capsules for size, colour, and weight across the treatment and PL conditions, the supplements were rendered physically indistinguishable, thereby effectively preserving the integrity of the blinding protocol. Participants and investigators involved in training supervision and outcome assessments were blinded to supplement allocation throughout the study. To evaluate the effectiveness of the blinding procedure, participants were asked at the end of the intervention to indicate whether they believed they had received Sb or PL. The randomised, placebo-controlled, double-blind design employed in the present study is widely recognised as the methodological gold standard for evaluating the efficacy of nutritional supplementation in exercise performance research.

### 2.5. Training intervention

All participants attended regular basketball training sessions four times per week during the preparatory phase of the season. These sessions focused on tactical and technical components of basketball performance, including passing, shooting, dribbling, defensive movements, and offensive strategies, as well as small-sided games and competitive simulations. Training sessions were conducted in the afternoon and lasted approximately 80–100 minutes. In addition to regular basketball practice, participants assigned to the training groups performed a structured SIT protocol three times per week for four weeks. To ensure consistency and adequate recovery, these sessions were scheduled on non-consecutive days (e.g. Saturday, Monday, and Wednesday) prior to the regular basketball training. The protocol consisted of four sets of ten maximal sprints lasting five seconds each, interspersed with 20-second active recovery periods involving walking or light jogging between repetitions. A three-minute passive recovery period was provided between sets, resulting in a total SIT duration of approximately 25 minutes per session [12,13]. The CON group participated only in the regular basketball training sessions without performing the SIT protocol or receiving supplementation. All SIT sessions were conducted on an indoor basketball court under the supervision of the research team. Adherence to the intervention was strictly monitored using mandatory attendance logs, and all participants included in the final analysis completed all SIT sessions. Participants wore their regular basketball training shoes, and all sessions were closely monitored by the strength and conditioning staff with a coach-to-player ratio of approximately 1:5 to ensure proper execution of the protocol and to provide verbal encouragement for maximal effort during each sprint.

### 2.6. Testing procedure

One week prior to the baseline measurements, all participants attended a standardised familiarisation session to minimise potential learning effects. During this session, participants practiced all performance assessments, including the Wingate anaerobic test and Yo-Yo IR1, jumping protocols, sprinting, and CODS tests, using identical equipment and procedures as in the actual trials. All assessments were performed during standardised testing sessions under controlled environmental conditions (temperature: 27–29 °C; relative humidity: 40–45%). Participants were instructed to follow consistent pre-testing guidelines before each session, including obtaining at least 8 hours of sleep, maintaining a carbohydrate-rich diet, and avoiding alcohol, caffeine, and other ergogenic substances. Compliance with these instructions was confirmed through verbal screening prior to testing. To reduce potential circadian influences, all evaluations were conducted at approximately the same time of day, and identical testing procedures were implemented during both the pre- and post-intervention assessments.

#### 2.6.1. Anthropometric

Participants’ anthropometric characteristics were assessed prior to testing. Standing height was measured to the nearest 0.1 cm using a wall-mounted stadiometer (SECA, Germany), with participants standing barefoot in an upright position. Body mass was recorded to the nearest 0.1 kg using a calibrated digital scale (BEURER, Germany), with participants wearing light clothing and no shoes.

#### 2.6.2. Jumping ability

Lower-body muscular power was evaluated using the CMVJ and the SLJ tests. Prior to testing, all participants completed a standardised warm-up consisting of light jogging, dynamic stretching, and several submaximal jumping trials. For the CMVJ, jump height was measured using a Vertec vertical jump device (Power Systems, Knoxville, TN, USA) installed on the basketball court. Participants stood upright beneath the apparatus and first performed a maximal reach with the dominant hand to determine standing reach height. They were then instructed to perform a rapid downward movement followed immediately by a maximal vertical jump without using arm swing. Participants were required to take off with both feet and land on the same spot without stepping forward. Three maximal CMVJ attempts were performed with 30 s of passive recovery between trials, and the highest jump height (cm) was recorded for subsequent analysis [9]. Horizontal muscular explosive power was assessed using the SLJ test. Participants stood behind a marked starting line with feet shoulder-width apart and were instructed to jump forward as far as possible using a countermovement and arm swing while taking off and landing with both feet simultaneously. The distance from the starting line to the nearest heel contact on landing was measured using a tape measure. Each participant performed three maximal attempts separated by 30 s of rest, and the longest distance (cm) was retained for analysis [9]. Previous studies have reported high test–retest reliability for both assessments, with intraclass correlation coefficients of 0.95 for CMVJ and 0.97 for SLJ [9,10].

#### 2.6.3. Sprinting and change of direction speed

Linear sprint and change-of-direction ability were assessed using the 20-m sprint test and the Illinois CODS test. For the 20-m sprint test, participants performed maximal sprints over a 20-m distance on an indoor basketball court. Sprint time was recorded using electronic timing gates (JBL Systems, Oslo, Norway) positioned at the start and finish lines, with accuracy to the nearest 0.01 s. Participants started from a standing position approximately 0.5 m behind the first timing gate to avoid early triggering. Following the two familiarisation trials, each participant completed three maximal sprint trials separated by 2 min of passive recovery. The fastest sprint time was retained for subsequent analysis [11]. The Illinois CODS test was used following the standard protocol described by Miller et al. Participants started from a prone position behind the starting line and completed the course by sprinting and weaving through a series of cones arranged according to the Illinois test layout (Figure 1). Time was recorded using the same electronic timing gate system positioned at the start and finish lines. After two familiarisation trials, participants performed three maximal attempts with 3 min of rest between trials. The best performance time was used for statistical analysis [32]. Previous research has demonstrated high reliability for these tests, with reported test–retest reliability coefficients of 0.93 for the 20-m sprint and 0.94 for the Illinois CODS test [11,32].

**Figure 1.**
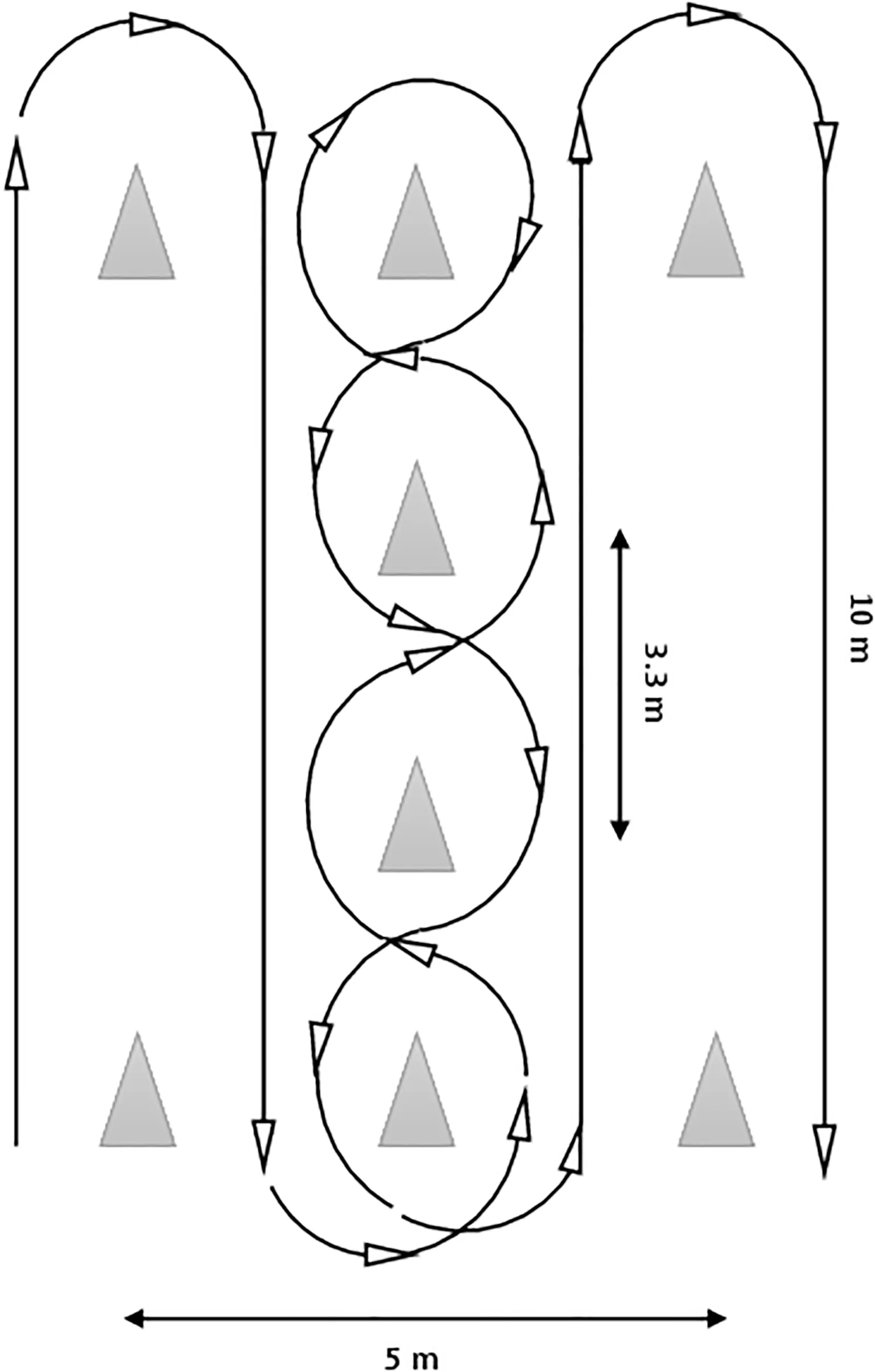
Illinois CODS procedure.

#### 2.6.4. Yo-Yo IR1

The Yo-Yo IR1 was used to evaluate participants’ intermittent aerobic endurance capacity. The test was conducted on an indoor basketball court according to the standard protocol described by Bangsbo [33]. Participants performed repeated 20-m shuttle runs at progressively increasing speeds dictated by audio signals, with a 10-s active recovery period between runs. The test was terminated when the participant failed to reach the line on two consecutive occasions or stopped due to exhaustion. The total distance covered was recorded as the final performance score (m).

#### 2.6.5. Wingate anaerobic power

The Wingate anaerobic power test was performed on a calibrated cycle ergometer (Ergomedics 874, Monark, Sweden). Participants initially completed a 5-minute standardised warm-up of low-intensity cycling to prepare the neuromuscular system for maximal exertion. Following the warm-up, participants were instructed to pedal at maximal speed for 30 seconds against a constant resistance of 0.075 kg·kg⁻^1^ of body mass. While higher resistance factors are occasionally used for elite athletes [34], the 0.075 coefficient was selected here as it remains the standardised protocol for team-sport athletes, ensuring comparability with existing performance data in trained basketball players [10]. To ensure maximal effort and mitigate potential variations in motivation, standardised “strong verbal encouragement” was provided throughout the 30-second bout by the same investigator. It is well-documented that such coaching interventions can significantly enhance subjective effort, mood state, and physical performance in athletic populations [35]. Peak power output (PPO) was recorded as the highest power output averaged over a 5-second window, while mean power output (MPO) represented the average power across the full duration. These metrics are established indicators of anaerobic capacity in high-intensity intermittent sports.

### 2.7. Diet control and monitoring of gastrointestinal tolerance to Sb supplementation

GI tolerance to sodium bicarbonate supplementation was monitored throughout the supplementation period. Participants were asked about symptoms such as nausea, bloating, abdominal discomfort, and related adverse effects during each supervised training session. However, a validated gastrointestinal distress questionnaire was not used; therefore, symptom assessment was based on informal participant interviews. Participants were encouraged to report any perceived GI disturbances to the research staff immediately after ingestion of the supplement or during subsequent sessions. This monitoring relied on self-disclosure rather than structured questionnaires or symptom logs, which introduced the possibility that mild or transient discomfort could have been underreported [36].

To minimise the potential influence of nutritional status on performance outcomes, basic dietary monitoring procedures were implemented. Participants were instructed to avoid the consumption of nutritional supplements or performance-enhancing substances throughout the study period. Dietary intake was assessed using a 3-day dietary record (two weekdays and one weekend day) at baseline to estimate habitual energy and macronutrient intake using Nutritionist Pro™ software (version IV). Participants were further instructed to maintain their usual dietary habits during the intervention and to avoid introducing new supplements or making substantial dietary changes during the study period.

Analysis of the dietary records indicated comparable caloric intake among participants, with the mean dietary composition approximating 20% protein, 60% carbohydrates, and 20% fat. Although continuous dietary monitoring was not performed throughout the entire intervention, these procedures were implemented to provide an estimate of habitual dietary intake and to reduce the likelihood that major dietary differences influenced the study outcomes.

### 2.8. Statistical analysis

All statistical procedures were performed using SPSS statistical software (version 21.0; SPSS Inc., Chicago, IL, USA). Data are presented as mean ± standard deviation (SD). Prior to the main analyses, the assumption of normality was examined using the Shapiro–Wilk test, and homogeneity of variances was verified with Levene’s test. To examine the effects of the intervention, a two-way repeated-measures analysis of variance (ANOVA) was conducted with time (pre vs. post) as the within-subject factor and group (SIT + 0.3 g·kg⁻^1^ Sb, SIT + 0.45 g·kg⁻^1^ Sb, SIT + PL, and CON) as the between-subject factor. When significant interactions or main effects were observed, Bonferroni-adjusted post hoc tests were applied to control for multiple comparisons and to reduce the risk of Type I error. Customised Excel spreadsheets were used to calculate all effect size (ES) statistics and their corresponding 95% confidence intervals (CIs). Hedge's g was specifically selected to calculate the ES for all measures, using the pooled standard deviation. ESs were interpreted according to the criteria proposed by Hopkins et al [37]: values < 0.2 were considered trivial, 0.2–0.6 small, 0.6–1.2 moderate, 1.2–2.0 large, 2.0–4.0 very large, and >4.0 nearly perfect. In addition, the percentage change for each variable was calculated using the following formula: (∆% = [(post − pre)/pre] × 100). Between-group differences in percentage changes were further analysed using a one-way ANOVA, followed by Bonferroni post hoc testing where appropriate. Blinding efficacy was evaluated by analysing participants’ allocation guesses at the end of the intervention. The distribution of correct and incorrect guesses across groups was compared using a chi-square test. In addition, binomial tests were conducted within each group to determine whether the proportion of correct guesses differed from chance level (50%). The level of statistical significance was set at *p* < 0.05 for all analyses.

## 3. Results

All participants successfully completed the assigned training and testing protocols, resulting in a 100% compliance rate confirmed through individual attendance records. No injuries related to training or testing occurred during the study period. No GI adverse effects were reported during supervised interviews; however, this tolerability outcome was based on informal symptom monitoring rather than a validated GI distress questionnaire. Blinding efficacy was assessed by asking participants to guess their treatment allocation at the end of the intervention. Correct guesses were reported by 6 of 10 participants in the 0.3 g·kg⁻^1^ Sb group, 7 of 10 in the 0.45 g·kg⁻^1^ Sb group, and 6 of 10 in the placebo/control group. The distribution of guesses did not differ significantly among groups (χ^2^ = 1.90, *p* = 0.387). Furthermore, within-group binomial tests indicated that the proportion of correct guesses did not differ significantly from chance (50%) in any group. These findings suggest that participant blinding was reasonably maintained under the study conditions.

### 3.1. Main effects

After the 4-week intervention, all SIT groups demonstrated significant improvements from pre- to post-testing (time effect, *p* = 0.001), with effect sizes ranging from small to large across all performance variables (Tables 2 and 3). In contrast, the CON group exhibited no significant changes between pre- and post-testing for any of the measured variables.

**Table 2.** Changes in the jumping and sprinting abilities as well as CODS performance from pre to post-training (mean ± SD).

Variables	Pre	Post	Hedge's g with 95 % CI	Group x Time interaction
**CMVJ (cm)**				
*SIT + 0.3 g Sb*	41.1 ± 2.7	42.8 ± 2.6*	0.61 (−0.28 to 1.51)	F_(3, 36)_ = 51.3, *p* = 0.001, η^2^_p_ = 0.811
*SIT + 0.45 g Sb*	41.9 ± 3.2	44.4 ± 3.1*	0.76 (−0.15 to 1.67)	
*SIT + PL*	41.0 ± 2.8	42.1 ± 2.6*	0.39 (−0.49 to 1.27)	
*CON*	41.2 ± 3.3	41.4 ± 3.3	NA	
**SLJ (cm)**				
*SIT + 0.3 g Sb*	272.4 ± 6.1	278.1 ± 5.7*	0.92 (0.00 to 1.85)	F_(3, 36)_ = 52.4, *p* = 0.001, η^2^_p_ = 0.814
*SIT + 0.45 g Sb*	272.5 ± 6.7	281.0 ± 6.8*	1.21 (0.25 to 2.16)	
*SIT + PL*	273.7 ± 6.9	277.2 ± 6.8*	0.49 (−0.40 to 1.38)	
*CON*	274.3 ± 7.1	274.8 ± 7.7	NA	
**20-m sprint (sec)**				
*SIT + 0.3 g Sb*	3.81 ± 0.30	3.69 ± 0.29*	−0.39 (−1.27 to 0.50)	F_(3, 36)_ = 22.1, *p* = 0.001, η^2^_p_ = 0.649
*SIT + 0.45 g Sb*	3.81 ± 0.20	3.64 ± 0.17*	−0.88 (−1.79 to 0.04)	
*SIT + PL*	3.82 ± 0.24	3.74 ± 0.21*	−0.34 (−1.22 to 0.54)	
*CON*	3.81 ± 0.27	3.80 ± 0.28	NA	
**Illinois CODS (sec)**				
*SIT + 0.3 g Sb*	18.54 ± 1.1	17.94 ± 0.9*	−0.57 (−1.47 to 0.32)	F_(3, 36)_ = 21.9, *p* = 0.001, η^2^_p_ = 0.645
*SIT + 0.45 g Sb*	18.52 ± 0.7	17.70 ± 0.6*	−1.21 (−2.16 to −0.25)	
*SIT + PL*	18.54 ± 1.1	18.14 ± 1.0*	−0.36 (−1.25 to 0.52)	
*CON*	18.50 ± 0.8	18.45 ± 0.7	NA	

^*^ denotes significant differences compared with Pre and CON (*p* < 0.05). η^2^_p_: Partial eta squared, representing the effect size for the group × time interaction.

**Table 3.** Changes in the Yo-Yo IR1 and anaerobic power performance from pre to post-training (mean ± SD).

Variables	Pre	Post	Hedge's g with 95 % CI	Group x Time interaction
**Yo-Yo IR1 (m)**				
*SIT + 0.3 g Sb*	1484 ± 118	1606 ± 112*	1.02 (0.08 to 1.95)	F_(3, 36)_ = 60.6, *p* = 0.001, η^2^_p_ = 0.835
*SIT + 0.45 g Sb*	1466 ± 147	1636 ± 114*	1.24 (0.28 to 2.19)	
*SIT + PL*	1470 ± 89	1546 ± 83*	0.85 (−0.07 to 1.76)	
*CON*	1482 ± 74	1502 ± 89	NA	
**PPO (w)**				
*SIT + 0.3 g Sb*	803 ± 55	872 ± 58*	1.17 (0.22 to 2.12)	F_(3, 36)_ = 64.7, *p* = 0.001, η^2^_p_ = 0.844
*SIT + 0.45 g Sb*	796 ± 61	892 ± 57*	1.56 (0.56 to 2.56)	
*SIT + PL*	781 ± 62	825 ± 64*	0.67 (−0.23 to 1.57)	
*CON*	805 ± 52	812 ± 51	NA	
**MPO (w)**				
*SIT + 0.3 g Sb*	468 ± 39	498 ± 42*	0.71 (−0.19 to 1.61)	F_(3, 36)_ = 48.2, *p* = 0.001, η^2^_p_ = 0.801
*SIT + 0.45 g Sb*	482 ± 31	529 ± 38*	1.30 (0.22 to 2.26)	
*SIT + PL*	471 ± 35	492 ± 38*	0.55 (0.34 to 1.44)	
*CON*	472 ± 48	479 ± 50	NA	

^*^ denotes significant differences compared with Pre and CON (*p* < 0.05). η^2^_p_: Partial eta squared, representing the effect size for the group × time interaction.

### 3.2. Group × time interaction effects

A significant group × time interaction was found for all performance variables (*p* = 0.001), indicating that changes over time differed significantly between the SIT groups and the CON group (Tables 2 and 3).

### 3.3. Sb vs. PL effects

Post hoc analyses revealed that the SIT + 0.3 g Sb and SIT + 0.45 g Sb groups demonstrated greater improvements than the SIT + PL group in the CMVJ (*p* = 0.011, 0.001), SLJ (*p* = 0.006, 0.001), 20-m sprint (*p* = 0.013, 0.001), Illinois CODS (*p* = 0.019, 0.001), Yo-Yo IR1 (*p* = 0.006, 0.001), PPO (*p* = 0.023, 0.001) and MPO (*p* = 0.037, 0.001) (Figures 2 and 3), respectively.

**Figure 2.**
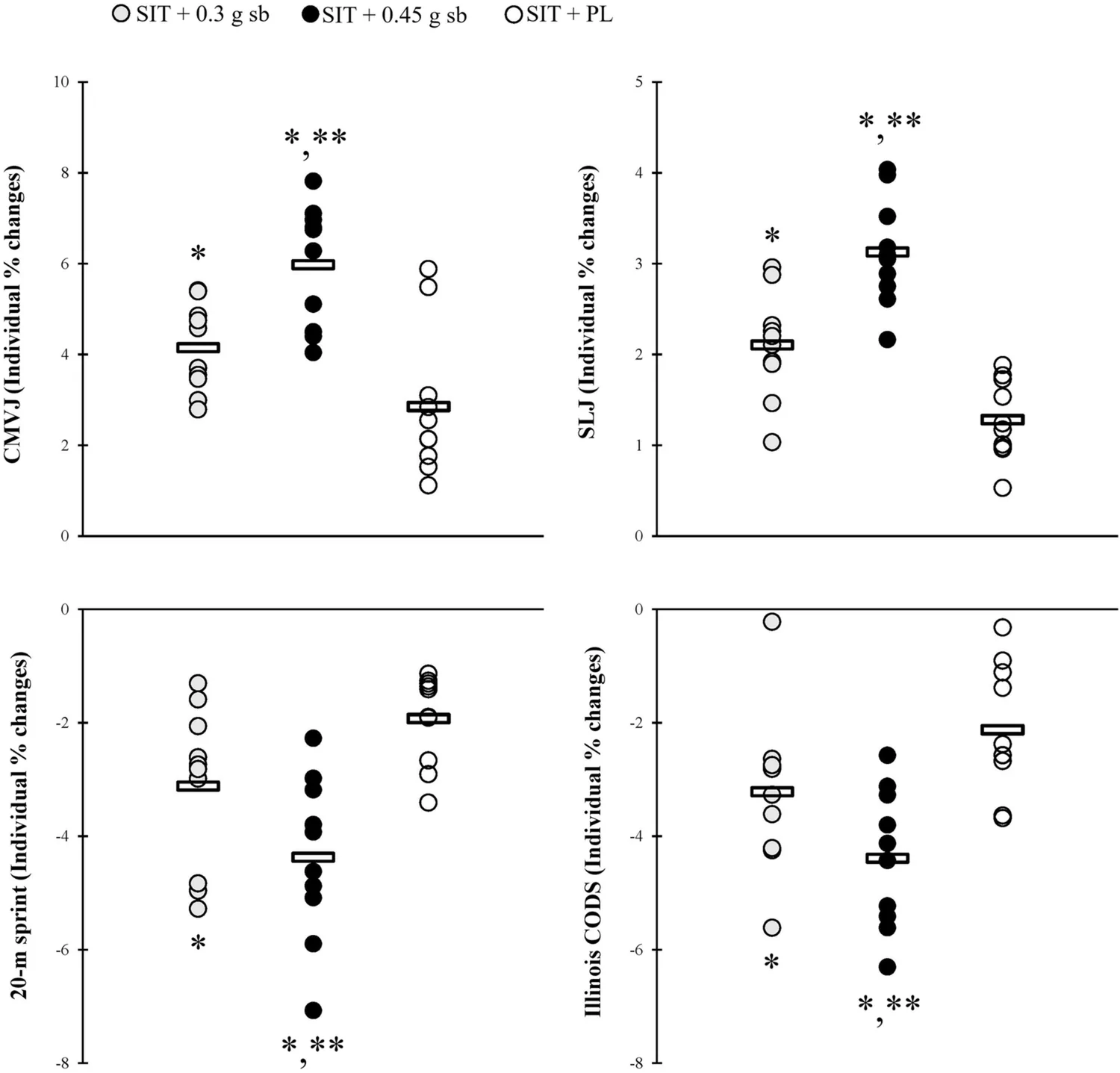
% changes in the jumping ability, sprint and CODS performance through the 4-week intervention period. *Denotes significant differences compared with the PL, **denotes significant differences compared with the SIT + 0.3 g Sb.

**Figure 3.**
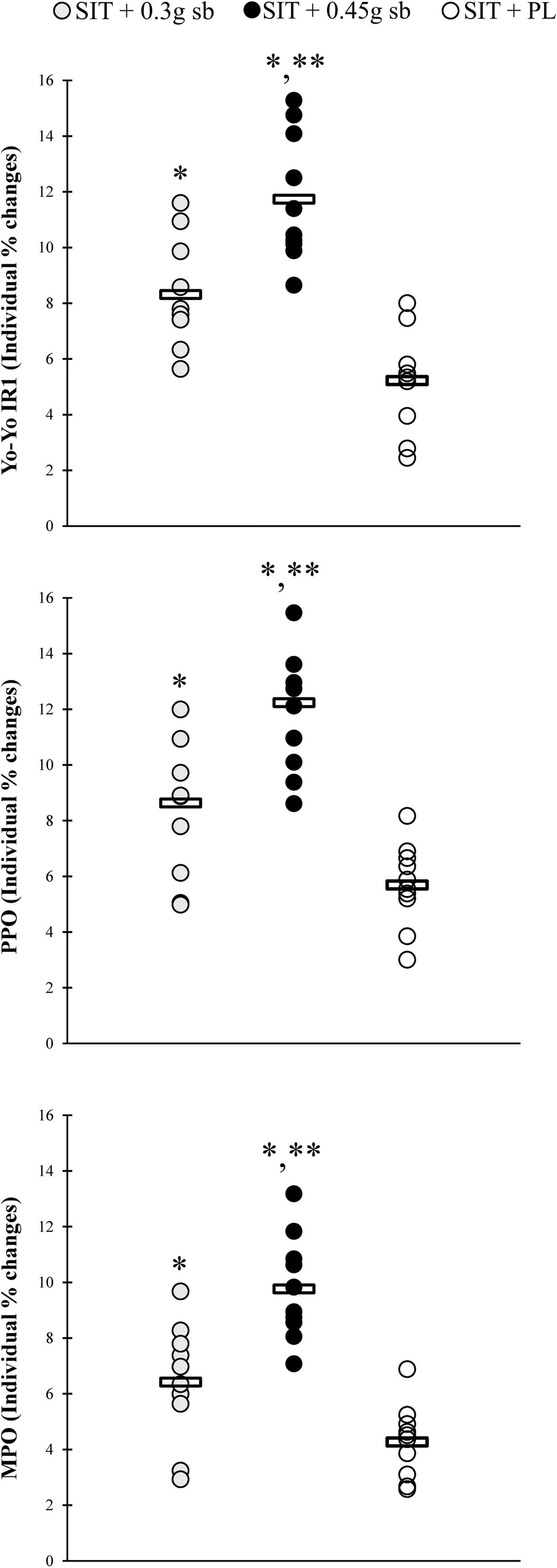
% changes in the Yo-Yo IR1 and anaerobic power performance through the 4-week intervention period. *Denotes significant differences compared with the PL, **denotes significant differences compared with the SIT + 0.3 g Sb.

### 3.4. Dose-dependent effects

When comparing the magnitude of adaptations, the SIT + 0.45 g Sb group demonstrated significantly greater improvements than the SIT + 0.3 g Sb group in CMVJ (5.9% vs. 4.1%, *p* = 0.001), SLJ (3.1% vs. 2.1%, *p* = 0.001), 20-m sprint (−4.1% vs. −3.1%, *p* = 0.001), Illinois CODS (−4.3% vs. −3.2%, *p* = 0.001), Yo-Yo IR1 (11.7% vs. 8.3%, *p* = 0.001), PPO (12.2% vs. 8.6%, *p* = 0.001), and MPO (9.7% vs. 6.4%, *p* = 0.001) (Figures 2 and 3).

## 4. Discussion

The purpose of the present study was to examine the dose-dependent effects of Sb supplementation combined with SIT on physical performance adaptations in trained basketball players during a short-term preparatory phase. The main findings were that: (1) four weeks of SIT significantly improved jumping performance, sprint speed, change-of-direction ability, intermittent endurance, and anaerobic power; (2) the addition of Sb supplementation produced greater improvements compared with PL; and (3) a higher dose of Sb (0.45 g·kg^−1^ BM) resulted in more pronounced improvements than the standard dose (0.3 g·kg^−1^ BM). Collectively, these findings suggest that extracellular buffering support during SIT may enhance training adaptations relevant to basketball performance.

### 4.1. Effects of SIT on performance adaptations

Consistent with previous literature [3,6,9–11], the present results showed that a four-week SIT programme significantly improved several physical performance variables in basketball players. SIT is characterised by repeated bouts of maximal or near-maximal exercise separated by short recovery periods, which impose considerable metabolic and neuromuscular stress [5]. Traditionally, these performance gains are attributed to rapid adaptations in both anaerobic and aerobic energy systems, including enhanced glycolytic enzyme activity, improved mitochondrial function, and increased neuromuscular efficiency [11,12]. However, beyond these cellular adaptations, it is essential to acknowledge the critical role of systemic physiological shifts. Recent evidence, such as the work by Sellami et al. [38], highlights that combined sprint-based training programmes can elicit significant haematological variations, including shifts in haemoglobin, haematocrit, and plasma volume. These alterations are pivotal for optimising systemic buffering capacity and enhancing metabolic clearance during high-intensity efforts. Such haematological adaptations likely complement enzymatic and mitochondrial improvements, collectively supporting the superior explosive power and intermittent endurance observed in our subjects. Therefore, the observed improvements in basketball-specific performance should be viewed as a synergistic result of both local muscular adaptations and systemic haematological refinements [6,10,11,38].

The improvements observed in CMVJ and SLJ performance are likely associated with neuromuscular adaptations such as increased motor unit recruitment, enhanced rate of force development, and improved intermuscular coordination [13]. Additionally, repeated high-intensity sprint efforts may improve the utilisation of the stretch–shortening cycle and increase muscle–tendon stiffness, thereby enhancing explosive force production during jumping tasks [15]. Similarly, improvements in 20-m sprint and Illinois CODS performance may reflect enhanced neuromuscular coordination, greater stride efficiency, and improved force transmission within the lower limb musculature [9–11].

The observed enhancement in Yo-Yo IR1 and anaerobic power performance further supports the effectiveness of SIT in improving intermittent endurance capacity and PPO as well as MPO [10,11]. The Yo-Yo test reflects an athlete’s ability to repeatedly perform high-intensity running efforts with brief recovery periods, a physiological demand that closely resembles the activity profile of basketball [33]. Adaptations such as increased mitochondrial density, improved oxidative enzyme activity, and enhanced oxygen utilisation likely contributed to the improved power and endurance performance in these athletes [13,15,39].

### 4.2. Additional effects of Sb supplementation

Beyond the benefits of SIT alone, the present findings demonstrate that Sb supplementation further enhanced performance adaptations across all measured variables compared with PL. These results are consistent with the established ergogenic role of Sb as an extracellular buffering agent resulting in more performance gains [40–42].

During high-intensity exercise, the rapid rate of glycolysis leads to the accumulation of hydrogen ions (H^+^), resulting in decreased intracellular pH and impaired muscle contractile function [4,5,43]. Elevated acidity within muscle cells can inhibit glycolytic enzyme activity, disrupt excitation–contraction coupling, and accelerate the onset of fatigue [5]. Sb supplementation is theoretically proposed to enhance the extracellular buffering capacity by increasing blood bicarbonate concentration and extracellular pH. This mechanism facilitates the efflux of H^+^ ions from active muscle fibres, which may help maintain intracellular acid–base balance during intense exercise, potentially leading to the performance enhancements observed in various studies [20,21,27,40,41]. By delaying the onset of metabolic acidosis, Sb supplementation may enable athletes to sustain higher training intensities during SIT sessions [40,41]. The ability to perform repeated high-intensity efforts with reduced fatigue may lead to greater total training efficacy and higher quality training stimuli [20]. Over time, this increased physiological stress can promote superior neuromuscular and metabolic adaptations, which may explain the greater improvements observed in the Sb groups [19]. However, it is important to acknowledge that a limitation of the present study is the absence of direct physiological markers, such as blood lactate, blood pH, and plasma HCO₃⁻ concentrations, to verify that the supplementation induced these specific metabolic alterations. Consequently, while our performance data are consistent with this buffering-enhancement hypothesis, it cannot mechanistically confirm the specific extracellular environment changes in the participants. Future research incorporating pre- and post-exercise blood chemistry analysis is warranted to definitively bridge the gap between Sb-induced buffering and the performance improvements in basketball-specific tasks.

### 4.3. Dose-dependent effects of Sb

An important finding of the present study is the clear dose-dependent response observed across all performance variables. Athletes who consumed 0.45 g·kg^−1^ BM Sb demonstrated significantly greater improvements than those receiving the 0.3 g·kg^−1^ BM dose. These results suggest that increasing Sb dosage may further elevate extracellular buffering capacity and enhance the removal of H⁺ ions from working muscles during high-intensity exercise [21,26,29].

The larger improvements observed in CMVJ, SLJ, sprint performance, and CODS in the higher-dose group may indicate that greater buffering capacity allows athletes to perform explosive movements with less metabolic disturbance during training [20]. Consequently, athletes may be able to sustain higher neuromuscular output during repeated sprint bouts, which could promote greater improvements in lower-body power and speed-related performance [40].

Similarly, the dose-dependent increases in PPO and MPO observed during the Wingate test suggest enhanced anaerobic metabolic capacity following the combined SIT and Sb intervention. Increased extracellular buffering may preserve glycolytic enzyme activity and maintain excitation–contraction processes during maximal efforts, thereby supporting greater ATP production via anaerobic pathways [27]. Additionally, improved acid–base balance may facilitate faster recovery between repeated high-intensity efforts during training sessions, contributing to greater improvements in anaerobic performance [28,29].

The greater improvement in Yo-Yo IR1 performance observed with the higher Sb dose highlights the potential contribution of enhanced buffering capacity to intermittent endurance performance. Sb supplementation primarily improves high-intensity exercise performance by increasing extracellular buffering capacity and facilitating H^+^ efflux from the working muscle [19]. Consequently, improved tolerance to metabolic acidosis during repeated high-intensity efforts may enable athletes to sustain intermittent running bouts for longer durations, thereby contributing to greater improvements in Yo-Yo IR1 performance [18]. The magnitude of improvement in Yo-Yo IR1 performance in the present study should also be interpreted in the context of the response observed in the PL group and the graded pattern across supplementation conditions. The SIT + PL group improved by approximately 5%, indicating that SIT alone produced a meaningful training adaptation over the 4-week intervention. However, larger improvements were observed in the Sb groups, with increases of 8.3% and 11.7% in the SIT + 0.3 g·kg⁻^1^ Sb and SIT + 0.45 g·kg⁻^1^ Sb groups, respectively. This progressive pattern suggests that Sb supplementation may have provided an additional stimulus that augmented the training response. From a physiological perspective, enhanced extracellular buffering may allow athletes to better tolerate the accumulation of H^+^ during repeated SIT sessions, potentially enabling the maintenance of higher exercise intensity and overall training quality. Over time, this may contribute to greater improvements in intermittent running performance. Nevertheless, because the evidence regarding chronic Sb supplementation and training adaptations remains inconsistent, the present findings should be interpreted cautiously and considered suggestive of a possible additive effect rather than definitive evidence of a dose-dependent training adaptation [19,20].

### 4.4. Limitations and future directions

Several limitations should be considered when interpreting the present findings. First, although the observed improvements in performance are consistent with the theoretical framework of Sb supplementation, the present study did not include direct physiological measurements capable of verifying the proposed extracellular buffering mechanism. Specifically, key biochemical markers such as blood pH, blood lactate concentration, and plasma bicarbonate levels were not assessed before or after exercise. Future studies should therefore incorporate repeated blood sampling before and after exercise bouts in order to quantify lactate accumulation, blood pH responses, and circulating bicarbonate levels, thereby providing stronger mechanistic evidence linking Sb supplementation to enhanced buffering capacity and improved high-intensity performance. In addition to the lack of biochemical markers, the potential contribution of systemic haematological responses should also be acknowledged. These physiological adjustments may influence oxygen transport, acid–base regulation, and metabolic by-product clearance during repeated high-intensity efforts. Another limitation concerns the relatively short duration of the intervention. The training and supplementation protocol lasted four weeks, which may be sufficient to induce short-term neuromuscular and metabolic adaptations but does not allow conclusions regarding the long-term sustainability of these responses. It remains unclear whether the improvements in sprint, power, and intermittent endurance performance would persist, plateau, or further increase during longer training cycles or throughout an entire competitive season. Moreover, the present sample consisted exclusively of male basketball players, which limits the generalisability of the findings to female athletes or to athletes from other sports with different physiological demands. Another methodological limitation relates to the assessment of GI tolerability. Although no gastrointestinal complaints were reported by the participants during the supplementation period, GI symptoms were monitored informally rather than assessed using a validated and standardised questionnaire. This is an important consideration because Sb supplementation is frequently associated with gastrointestinal disturbances such as nausea, abdominal discomfort, bloating, or diarrhoea, particularly when administered in large acute doses. In the present study, the total dose of Sb was divided into five equal servings, a strategy that may reduce the likelihood of GI distress by slowing GI absorption and minimising osmotic effects. Future studies should therefore employ standardised GI symptom questionnaires to more rigorously evaluate the tolerability and practical applicability of different dosing strategies. Finally, dietary intake represents another factor that may influence buffering-related supplementation outcomes. Nutritional intake during the intervention was assessed using a 3-day dietary record, which provided a general estimate of participants’ habitual dietary patterns. However, this method may not fully capture day-to-day dietary variability across the entire four-week intervention period. Although participants were instructed to maintain their habitual diet and baseline analyses indicated no systematic differences between groups, more comprehensive nutritional monitoring—such as repeated dietary records or controlled dietary protocols—would strengthen internal validity in future supplementation studies. Taken together, these limitations highlight the need for more comprehensive experimental designs in future research. Specifically, integrating biochemical markers of acid–base balance, haematological measurements, detailed gastrointestinal tolerance assessments, and more extensive dietary monitoring would provide a more complete understanding of the physiological mechanisms through which sprint interval training and sodium bicarbonate supplementation influence performance adaptations in basketball players.

## 5. Conclusion

In summary, the present study suggests that SIT is associated with improvements in jumping ability, sprint performance, CODS, intermittent endurance, and anaerobic power in trained basketball players. Sb supplementation appears to further enhance these adaptations, with a higher dose of 0.45 g·kg⁻^1^ BM demonstrating potentially greater benefits than the standard 0.3 g·kg⁻^1^ BM dosage. From a practical perspective, coaches and practitioners can strategically implement this combined intervention—utilising a split-dosing strategy—during short-term, high-intensity preparatory phases (e.g. a 4-week pre-season) to accelerate physical readiness. This approach serves as a time-efficient modality to optimise anaerobic performance and bio-motor abilities in basketball athletes.
